# Quantifying Magnetic Sensitivity of Radical Pair Based Compass by Quantum Fisher Information

**DOI:** 10.1038/s41598-017-06187-y

**Published:** 2017-07-19

**Authors:** Li-Sha Guo, Bao-Ming Xu, Jian Zou, Bin Shao

**Affiliations:** 1 School of Physics, Beijing Institute of Technology, Beijing, 100081 China; 2 School of Physics, Qufu Normal University, Qufu, 273165 China

## Abstract

The radical pair (RP) based compass is considered as one of the principal models of avian magnetoreception. Different from the conventional approach where the sensitivity of RP based compass is described by the singlet yield, we introduce the quantum Fisher information (QFI), which represents the maximum information about the magnetic field’s direction extracted from the RP state, to quantify the sensitivity of RP based compass. The consistency between our results and experimental observations suggests that the QFI may serve as a measure to describe the sensitivity of RP based compass. Besides, within the framework of quantum metrology, we give two specific possible measurement schemes and find that the conventional singlet yield is corresponding to the measurement of total angular momentum. Moreover, we show that the measurement of fluctuation of the total magnetic moment is much more accurate than the singlet yield measurement, and is close to the optimal measurement scheme. Finally, the effects of entanglement and decoherence are also discussed in the spirit of our approach.

## Introduction

Recent evidence suggests that some unique features of quantum mechanics can be harnessed to enhance biological functions in a large variety of living organisms, e.g., in natural selection^1^, olfaction sense^2, 3^, enzymatic reactions^4, 5^, photosynthetic light harvesting^6, 7^, avian magnetoreception^8–30^, etc., which indicates that quantum biology has been entering a new stage^31–33^. As one of the principal models of avian magnetoreception, the radical pair (RP) mechanism^9–12^, based on singlet-triplet transitions due to the anisotropic hyperfine (HF) interaction, suggests that migratory birds depend on the photoinduced RPs created in ocular cryptochrome proteins for navigation, which has been supported by intensive evidences and behavioral experiments with birds^34–40^. Due to the quantum mechanical nature of RP model, a growing interest in understanding the function of avian magnetoreception has extended from chemists, biologists to physicists, by using the rich fruits in the field of quantum information such as quantum coherence and entanglement^13–20^. However, the essence of avian magnetoreception is to detect the geomagnetic field, i.e., to determine the direction of geomagnetic field. This makes us to recall the quantum metrology^41–43^, which has primarily been developed to find the fundamental limit to precision of estimating an unknown parameter, with the ultimate precision given by the quantum Fisher information (QFI) according to the quantum Cramér-Rao bound^44^. Up to now, quantum parameter estimation, as a fundamental and important subject in physics, has been applied to various aspects such as gravitational wave detectors^45, 46^, frequency spectroscopy^47, 48^, interferometry^49, 50^, atomic clocks^51, 52^, thermometry^53, 54^ and even in the magnetic field sensing^55–57^ (avian magnetoreception essentially belongs to this field). In fact, there has been a few relevant works which tried to apply the concepts and methods developed in quantum metrology to the field of avian magnetoreception. For instance, within the framework of quantum metrology, Jianming Cai *et al*. have quantified global quantum coherence with a quantum interferometer, and correlated it with the function of chemical magnetoreception^15^. Moreover, ref. 27 has revealed new magnetic-field effects conveyed by the Groenewold information extracted during the RP reaction. And very recently, we have noticed that ref. 28 introduced the tools of quantum metrology to put formal and fundamental limit to the precision of estimating magnetic field intensity of RP reactions.

In this paper, we apply quantum metrology to the RP based compass, and use the quantum Fisher information (QFI), which represents the maximum information about the geomagnetic field direction extracted from the RP state, to quantify its magnetic sensitivity (i.e., the precision of estimating magnetic field direction). Such an approach allows us to establish a quantitative connection between the performance of RP based compass and QFI. In the context of RP model, we first derive a statistical average state (i.e., a steady state) of RP, then calculate the QFI of this state and finally compare the results with the relevant experimental results. The consistency between the behavior of QFI and the experimental results corroborates our approach and suggests that the QFI may serve as a measure to describe the sensitivity of RP based compass. Besides, within the framework of quantum metrology, we shed light on an intriguing connection between the conventional singlet yield^10, 11^ and a concrete measurement scheme of our approach.

## Results

### Model

In the RP based compass, each photoinduced RP has a spatially separated electron pair coupled to an external magnetic field **B** and a few nuclei. Generally it is believed that only one of the electrons interacts with the nuclei with an anisotropic HF coupling and the other is free^8^. Thus this provides asymmetry and leads to singlet-triplet transition required for the directional sensitivity. In this paper, we only consider the simple case of one nuclear spin and the corresponding Hamiltonian for each RP is^17–22^

1 $$H=\gamma {\bf{B}}\cdot ({\hat{S}}_{1}+{\hat{S}}_{2})+\hat{I}\cdot {\bf{A}}\cdot {\hat{S}}_{2},$$

where ${\hat{S}}_{i}=({\sigma }_{x},{\sigma }_{y},{\sigma }_{z})$ are the electronic spin operators (*i* = 1, 2), and $\hat{I}$ is the nuclear spin operator. **B** is the external magnetic field around the RP, and *γ* = $\frac{1}{2}$ *μ*_0_ *g* the gyromagnetic ratio with *μ*_0_ being Bohr’s magneton and *g* = 2 the *g* factor. It should be noted that the factor 1/2 in the gyromagnetic ratio accounts for the fact that we use Pauli matrices (*σ*_*x*_, *σ*_*y*_, *σ*_*z*_) instead of the spin operator (*S*_*x*_, *S*_*y*_, *S*_*z*_). **A** is the HF tensor which couples the nuclear spin and electron 2 with a diagonal form **A** = *diag*(*A*_*x*_, *A*_*y*_, *A*_*z*_), and we assume an axially symmetric (or cigar-shaped) HF tensor, i.e., *A*_*z*_ > *A*_*x*_ = *A*_*y*_. The RP density matrix at time *t* can then be described as

2 $${\rho }_{s}(t)={{\rm{Tr}}}_{I}[U(t)\rho \mathrm{(0)}{U}^{\dagger }(t)],$$

where *U*(*t*) is the evolution operator corresponding to the Hamiltonian Eq. (1), and Tr_*I*_[⋅] means taking the trace over the nucleus. *ρ*(0) = *ρ*_*s*_(0) ⊗ *ρ*_*I*_(0) is the initial state of two electrons and one nucleus, and generally the nucleus is considered to be initially in a complete mixed state, i.e., ${\rho }_{I}\mathrm{(0)}={\mathbb{I}}/2$.

Firstly, we assume that in the RP based compass, all the RPs are identical and in the same initial state. Due to the continuous optical excitation, the creation of each RP is entirely accidental and its decay is also random. However, with respect to all the RPs, they would be in a steady state. In what follows, we would derive a statistical average state of RP to describe this steady state. To be more specific, choosing an arbitrary fixed time to see (here we set the fixed time as the reference time, denoted as *t*′ = 0), the RPs at the reference time (*t*′ = 0) are constituted of those evolved from different time *t*′ (*t*′ < 0), i.e., the moment of RP formation. It is reasonable to assume that in time regime *t*′ ~ *t*′ + *dt*′, the number of RPs created by optical excitation is a constant which is not dependent on the specific time *t*′, denoted by Δ*M*. And the number of them which still exist (not decay) at the reference time is *d*Δ*M*(*t*′) = Δ*Mf*(*t*′)*dt*′, where *f*(*t*′) ≡ *k* exp(−*k*|*t*′|), with *k* being the recombination rate^58^. In other words, for each RP created by optical excitation in time regime *t*′ ~ *t*′ + *dt*′, its existing probability at the reference time is

3 $$P(t^{\prime} )=\frac{d{\rm{\Delta }}M(t^{\prime} )}{{\rm{\Delta }}M}=f(t^{\prime} )dt^{\prime} ,$$

and the corresponding state at the reference time (*t*′ = 0) is described as *ρ*_*s*_(*t*′) which is evolved from the time regime *t*′ ~ *t*′ + d*t*′. Due to the fact that each RP is subjected to the optical excitation randomly, at the reference time, the state of the RP would be consisted of a large number of states evolved from different time *t*′ with a corresponding weight *P*(*t*′). As a result, we can obtain a statistical average state (i.e., the steady state) of RP:

4 $${\bar{\rho }}_{s}={\int }_{-\infty }^{0}f(t^{\prime} ){\rho }_{s}(t^{\prime} )dt^{\prime} ={\int }_{0}^{\infty }f(t){\rho }_{s}(t)dt,$$

where in the second equation, we have replaced the integration variable *t*′ with *t* = −*t*′, and accordingly *ρ*_*s*_(*t*′) is equal to *ρ*_*s*_(*t*) defined in Eq. (2). It is noted that ${\int }_{-\infty }^{0}f(t^{\prime} )dt^{\prime} ={\int }_{0}^{\infty }f(t)dt=1$.

Here we make a brief comment that this statistical average state of RP ${\bar{\rho }}_{s}$ contains all the information about the magnetic field’s direction, so obtaining this state is a critical step to further extracting information from the RP system. In the following sections, we would introduce how to use the QFI that represents the maximum information extracted from ${\bar{\rho }}_{s}$ to quantify the magnetic sensitivity of RP based compass, and how to extract information from ${\bar{\rho }}_{s}$ by means of some concrete measurement schemes.

### Magnetic sensitivity quantified by QFI

In this section, we would use the QFI to quantify the magnetic sensitivity of RP based compass by means of quantum parameter estimation theory (see Methods), and compare our QFI results with two typical behavioral experiments with birds.

#### Effect of external magnetic field on QFI

**Figure 1:**
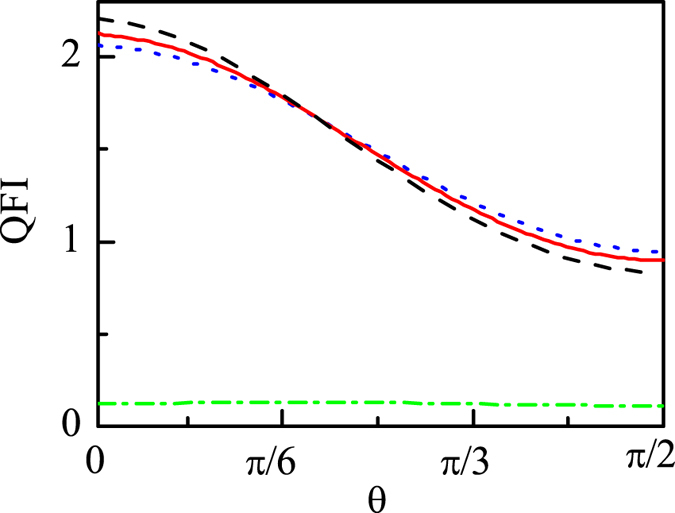
The QFI as a function of the direction angle *θ* without the oscillating field (${{\rm{B}}}_{{\rm{0}}}=46\,\mu$T (red solid line), ${{\rm{B}}}_{{\rm{0}}}=59.8\mu$T (black dashed line), and ${{\rm{B}}}_{{\rm{0}}}=32.2\mu$T (blue dotted line)), and with the oscillating field ${{\rm{B}}}_{{\rm{rf}}}=150$ nT and ${{\rm{B}}}_{{\rm{0}}}=46\mu$T (green dash dotted line). ${A}_{z}=6\gamma \times 46\mu$T, ${A}_{x}={A}_{y}=0$, $k={10}^{4}{s}^{-1}$.

In what follows we would calculate the QFI of the steady state ${\bar{\rho }}_{s}$ of RP according to the quantum parameter estimation theory (see Methods). In the main text, ${A}_{x}={A}_{y}=0$ (and the case ${A}_{x}={A}_{y}\ne 0$ is considered in Sec. B of the supplementary materials). Generally, the geomagnetic field can be described as

5 $${{\bf{B}}}_{{\bf{0}}}={{\rm{B}}}_{{\rm{0}}}(\sin \,\theta \,\cos \,\varphi ,\,\sin \,\theta \,\sin \,\varphi ,\,\cos \,\theta ),$$

where ${{\rm{B}}}_{{\rm{0}}}$ is the intensity of the geomagnetic field, and *θ* and $\varphi$ describe the orientation of the geomagnetic field to the basis of HF tensor. The axial symmetry of HF tensor allows us to set *ϕ* = 0 and focus on *θ* in the range $\mathrm{[0},\pi /\mathrm{2]}$ without loss of generality, and *θ* is the parameter to be estimated for the RP based compass. And then we can calculate the QFI of the steady state ${\bar{\rho }}_{s}$ of RP under the influence of the geomagnetic field, and in this case, ${\bf{B}}={{\bf{B}}}_{{\bf{0}}}$ in Eq. (1). It has been shown that *k* should the order of ${10}^{4}{s}^{-1}$ in different scenarios^20, 21, 23^, so in this paper we let $k={10}^{4}{s}^{-1}$ and will discuss the validity of it in terms of QFI below. When $k={10}^{4}{s}^{-1}$, for an arbitrary initial state of RP ${\rho }_{s}\mathrm{(0)}$, an approximate expression of QFI of the steady state ${\bar{\rho }}_{s}$ can be obtained, by making a strong HF coupling approximation, i.e., ${A}_{z}\gg \gamma {{\rm{B}}}_{{\rm{0}}}$ (the detailed derivation of QFI can be seen in Sec. A.1 of the supplementary materials):

6 $${\rm{QFI}}\approx \sum _{i\mathrm{=0}}^{1}{\rm{Re}}{[{\rho }_{i}^{12}]}^{2}(\frac{1}{{\rho }_{i}^{11}}+\frac{1}{{\rho }_{i}^{22}})+\frac{{({\rho }_{i}^{11}-{\rho }_{i}^{22})}^{2}}{{\rho }_{i}^{11}+{\rho }_{i}^{22}},$$

where ${\rho }_{1}^{ij}=\langle {\varphi }_{i}|\langle \mathrm{1|}{\rho }_{s}\mathrm{(0)|}{\varphi }_{j}\rangle \mathrm{|1}\rangle$, and ${\rho }_{0}^{ij}=\langle {\varphi }_{i}|\langle \mathrm{0|}{\rho }_{s}\mathrm{(0)|}{\varphi }_{j}\rangle \mathrm{|0}\rangle$, with $\mathrm{|0}\rangle$ ($\mathrm{|1}\rangle$) and $|{\varphi }_{i}\rangle$ ($i=1,2$) being the eigenstates of ${\sigma }_{z}$ of electron 2 and Hamiltonian of electron 1, i.e., ${H}_{1}=\gamma {{\bf{B}}}_{{\bf{0}}}\cdot {\hat{S}}_{1}$, respectively, and ${\rm{Re}}[{\rho }_{i}^{12}]$ represents the real part of ${\rho }_{i}^{12}$. From Eq. (6) we can see that for any given initial state ${\rho }_{s}\mathrm{(0)}$, the QFI of the steady state ${\bar{\rho }}_{s}$ is not dependent on ${{\rm{B}}}_{{\rm{0}}}$, which implies that the change of the intensity of external magnetic field ${{\rm{B}}}_{{\rm{0}}}$ would not disorient the bird permanently. This is consistent with the experimental result that bird can adapt to different magnetic field intensities^35–37^. Furthermore, without making any approximation, we numerically plot the QFI for different magnetic field intensities with the RP initial state being the singlet state $|S\rangle =\frac{1}{\sqrt{2}}\mathrm{(|10}\rangle -\mathrm{|01}\rangle )$ in Fig. 1 as an example. We can see from Fig. 1 that the $\mathrm{30 \% }$ weaker (32.2 *μ*T) and stronger (59.8 *μ*T) fields^35^ than the geomagnetic field (46 *μ*T) have almost no influences on the value of QFI, that is, bird would not disorient when the intensity of magnetic field is decreased or increased by about 30% of that of geomagnetic field.

#### Effect of weak oscillating field on QFI

We now proceed to investigate the influence of an additional weak resonant oscillating field on the QFI, and in this case, ${\bf{B}}={{\bf{B}}}_{{\bf{0}}}+{{\bf{B}}}_{{\bf{r}}{\bf{f}}}$ in Eq. (1) with

7 $${{\bf{B}}}_{{\bf{r}}{\bf{f}}}={{\rm{B}}}_{{\rm{rf}}}\,\cos \,wt(\sin \,\alpha \,\cos \,\beta ,\,\sin \,\alpha \,\sin \,\beta ,\,\cos \,\alpha ),$$

where ${{\rm{B}}}_{{\rm{rf}}}$ is the strength of oscillating field with frequency $\omega =2\gamma {{\rm{B}}}_{{\rm{0}}}$ being resonant with the free electron. *α* and *β* represent the direction of oscillating field with respect to the basis of HF tensor. Due to the axial symmetry of HF tensor we set *β* = 0. Firstly, we consider $\alpha =\theta +\pi /2$, i.e., the weak oscillating field is perpendicular to Earth’s magnetic field. In this case, when $k={10}^{4}{s}^{-1}$, for an arbitrary initial state of RP ${\rho }_{s}\mathrm{(0)}$, we can also obtain an approximate expression of QFI of the steady state ${\bar{\rho }}_{s}$, by making a strong HF coupling approximation (see Sec. A.2 of the supplementary materials for a detailed derivation):

8 $${\rm{QFI}}\approx \sum _{i=0}^{1}\frac{{k}^{4}{\rm{Re}}{[{\rho }_{i}^{12}]}^{2}}{{({k}^{2}+{(\gamma {{\rm{B}}}_{{\rm{rf}}})}^{{\rm{2}}})}^{2}}(\frac{1}{{P}_{i}^{11}}+\frac{1}{{P}_{i}^{22}})+\frac{{({P}_{i}^{11}-{P}_{i}^{22})}^{2}}{{P}_{i}^{11}+{P}_{i}^{22}},$$

where ${P}_{i}^{jj}={\rho }_{i}^{jj}+{(-\mathrm{1)}}^{j}{\chi }_{i}$, with ${\rho }_{i}^{jj}$ having been defined below Eq. (6), ${\chi }_{i}=\frac{{(\gamma {{\rm{B}}}_{{\rm{rf}}})}^{{\rm{2}}}}{\mathrm{2(}{k}^{2}+{(\gamma {{\rm{B}}}_{{\rm{rf}}})}^{{\rm{2}}})}({\rho }_{i}^{11}-{\rho }_{i}^{22})-$ $\frac{\gamma {{\rm{B}}}_{{\rm{rf}}}k}{({k}^{2}+{(\gamma {{\rm{B}}}_{{\rm{rf}}})}^{{\rm{2}}})}{\rm{Im}}[{\rho }_{i}^{12}]$ ($i=0,1$, $j=1,2$), and ${\rm{Im}}[{\rho }_{i}^{12}]$ represents the imaginary part of ${\rho }_{i}^{12}$. Through our calculation, we obtain that for any given initial state ${\rho }_{s}\mathrm{(0)}$, when $\gamma {{\rm{B}}}_{{\rm{rf}}}=0$ (without the oscillating field), Eq. (8) reduces to Eq. (6); when $\gamma {{\rm{B}}}_{{\rm{rf}}}\gg k$, QFI ≈ 0, which implies that the weak resonant oscillating field can completely disorient the bird.

**Figure 2:**
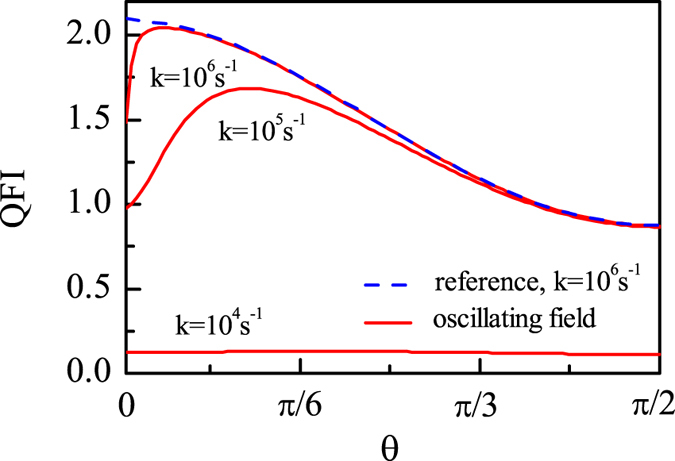
The QFI as a function of direction angle *θ* with a weak resonant oscillating field perpendicular to Earth’s magnetic field. ${A}_{z}=6\gamma \times 46\mu$T and ${A}_{x}={A}_{y}=0$. The blue dashed line provides a reference of QFI without the oscillating field for ${{\rm{B}}}_{{\rm{0}}}=46\mu$T (The reference is independent of the recombination rate *k* when $k\le {10}^{7}{s}^{-1}$). The red solid lines represent the QFI when a 150nT resonant oscillating field is applied.

In what follows, without making any approximation, we would discuss the appropriate order of $k$ in terms of QFI, by considering the experimental result that a weak resonant oscillating field (to be conservative, we take the field strength ${{\rm{B}}}_{{\rm{rf}}}$ = 150 nT) perpendicular to Earth’s magnetic field can disrupt the bird completely^38–40^. Here we also take the singlet state $|S\rangle$ as the initial state of RP as an example, and our numerical results are shown in Fig. 2. It can be seen that when $k={10}^{6}{s}^{-1}$, the QFI is almost immune to the oscillating field, and when $k={10}^{5}{s}^{-1}$, the QFI with the oscillating field is reduced to some extent compared with that without the oscillating field, but we are not sure whether this reduction of QFI can disrupt the birds or not. However, when $k={10}^{4}{s}^{-1}$, the value of QFI is highly reduced when the weak resonant oscillating field is applied. And we can also see from Fig. 1 that the weak resonant oscillating field (the green dash dotted line) can reduce the QFI significantly when $k={10}^{4}{s}^{-1}$. Thus it is safe to say that if the oscillating field is to disorient the bird, it might be approximately $k={10}^{4}{s}^{-1}$, which is in accordance with the previous works by means of conventional approach^20, 21, 23^. Besides, we find that the oscillating field parallel to Earth’s magnetic field does not affect the value of QFI, which is consistent with the experimental results^38–40^.

**Figure 3:**
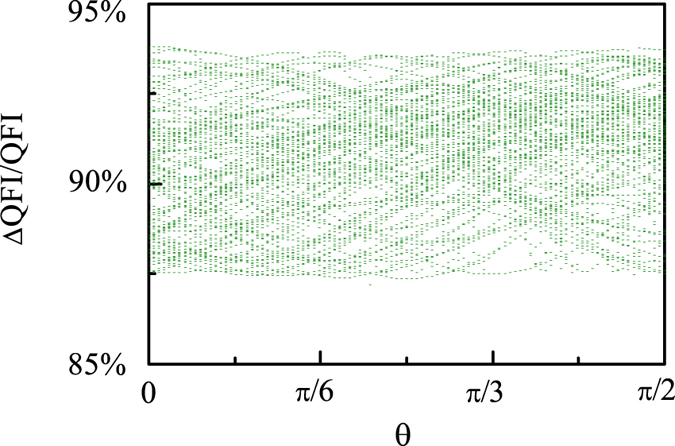
The percent decrease of QFI when a weak resonant oscillating field orthogonal to the geomagnetic field is applied, i.e., ${\rm{\Delta }}\mathrm{QFI}/\mathrm{QFI}$, as a function of the direction angle *θ* for 100 randomly sampled initial states of RP with ${A}_{z}=6\gamma \times 46\mu$T, ${A}_{x}={A}_{y}=0$, ${{\rm{B}}}_{{\rm{0}}}=46\mu$T, ${{\rm{B}}}_{{\rm{rf}}}=150$ nT and $k={10}^{4}{s}^{-1}$.

In addition, we would numerically show that when $k={10}^{4}{s}^{-1}$, for an arbitrary initial state of RP, a weak resonant oscillating field orthogonal to the geomagnetic field can highly reduce the value of QFI of the steady state ${\bar{\rho }}_{s}$. Specifically, we randomly sample 100 initial states and plot in Fig. 3 the corresponding percent decreases of QFI, i.e., ${\rm{\Delta }}\mathrm{QFI}/\mathrm{QFI}\equiv \frac{{\mathrm{QFI}(B}_{{\rm{rf}}}=0)-{\mathrm{QFI}(B}_{{\rm{rf}}}={\rm{150}}\,\mathrm{nT})}{{\mathrm{QFI}(B}_{{\rm{rf}}}=0)}$, as a function of *θ*. The results without making any approximation show that for all the sampled initial states, ΔQFI/QFI is larger than 87%, which implies that a weak resonant oscillating field orthogonal to the geomagnetic field can completely disorient the bird for an arbitrary initial state of RP.

Finally, it should be emphasized that the above two experimental results, which we have compared with our QFI results, have been previously explained by others from the point of singlet yield^17, 20, 21^. Here we reinterpret the experimental results in terms of QFI which can make them more clear and can also check the validity of our QFI approach.

### Possible implementations for RP based compass

Up to now, we have used the QFI to quantify the magnetic sensitivity of RP based compass, but it is only an upper bound of precision. In fact, there may exist several possible implementations and what specific kind of implementation is adopted in avian compass is not clear for us, despite of the prevailing view that the external magnetic field information can be recorded by the singlet yield^9–11^. When a specific POVM measurement, corresponding to an observable $\hat{O}$, has been performed, the unknown parameter *θ* can be estimated from the mean value of $\hat{O}$, with the precision given by the standard error propagation formula ${{\rm{\Delta }}}^{2}\theta =\frac{{{\rm{\Delta }}}^{2}\hat{O}}{|d\langle \hat{O}\rangle /d\theta {|}^{2}}$ ^59, 60^, where ${{\rm{\Delta }}}^{2}\hat{O}$ and $\langle \hat{O}\rangle$ represent the variance and mean value of the observable $\hat{O}$ obtained for ${\bar{\rho }}_{s}$, respectively. Given that the initial state of RP is in the singlet state $|S\rangle$, we give two possible implementations as examples here: they are the measurement of total angular momentum, and that of the square of magnetic moment, respectively.

**Figure 4:**
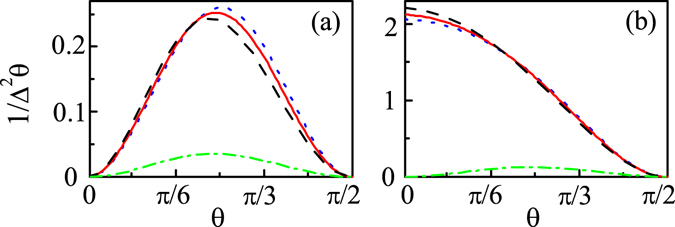
1/^2^Δ*θ* as a function of the direction angle *θ* for measuring (**a**) ${\hat{S}}^{2}$ and (**b**) ${\hat{S}}_{z}^{2}$ without the oscillating field (B_0_ = 46*μ*T (red solid line), B_0_ = 59.8 *μ*T (black dashed line), and ${{\rm{B}}}_{{\rm{0}}}=32.2\mu$T (blue dotted line)), and with the oscillating field B_rf_ = 150 nT and ${{\rm{B}}}_{{\rm{0}}}=46\mu$T (green dash dotted line). For both (**a**) and (**b**) the initial state of RP is the singlet state with ${A}_{z}=6\gamma \times 46\mu$T, ${A}_{x}={A}_{y}=0$ and $k={10}^{4}{s}^{-1}$.

Firstly, we give the measurement of total angular momentum, i.e., $\hat{O}={\hat{S}}^{2}={({\hat{S}}_{1}+{\hat{S}}_{2})}^{2}$. Here it should be noted that $\langle {\hat{S}}^{2}\rangle =\mathrm{2(1}-{P}_{S})$, with ${P}_{S}\equiv \langle S|{\bar{\rho }}_{s}|S\rangle$ representing the probability that the RP is found in the singlet state $|S\rangle$, besides, it can be seen from Eq. (4) that $\langle S|{\bar{\rho }}_{s}|S\rangle ={\int }_{0}^{\infty }f(t)\langle S|{\rho }_{s}(t)|S\rangle dt\equiv {{\rm{\Phi }}}_{S}$ ^17–21^. Thus, the signal contrast ${D}_{s}={{\rm{\Phi }}}_{max}-{{\rm{\Phi }}}_{min}$ ^15–18^ (i.e., the difference between the maximum and the minimum singlet yields along all the directions), which is conventionally used as a measure to quantify the magnetic sensitivity of avian compass, is actually corresponding to the measurement of ${\hat{S}}^{2}$. In other words, the conventional singlet yield is just one of the several possible measurement schemes of our approach. Moreover, through our calculations, we find that Δ^2^ *θ* is equal to the inverse of the classical Fisher information $\mathrm{1/}{\rm{F}}$. Although the signal contrast ${D}_{s}$ and the classical Fisher information F(1/${{\rm{\Delta }}}^{2}\theta$) both describe the magnetic sensitivity of avian compass, F(1/Δ^2^ *θ*), which denotes the maximum information about $\theta$ extracted from the steady state of RP for this measurement scheme, is more accurate and can better reflect the essence of avian magnetoreception than ${D}_{s}$. Our numerical results of $\mathrm{1/}{{\rm{\Delta }}}^{2}\theta$ are shown in Fig. 4(a), and we can see that the 30% stronger and weaker fields than Earth’s magnetic field almost have no influences on the value of 1/Δ^2^ *θ*, however, a weak resonant oscillating field perpendicular to Earth’s magnetic field reduces the value of 1/Δ^2^ *θ* dramatically.

Next, we give the measurement of square of magnetic moment, i.e., $\hat{O}={\hat{S}}_{z}^{2}={({\hat{S}}_{1z}+{\hat{S}}_{2z})}^{2}$ with ${\hat{S}}_{iz}$ being the *z* component of spin angular momentum of the $i$ th electron ($i=1,2$). Due to $\langle {\hat{S}}_{z}\rangle \mathrm{=0}$, the measurement of ${\hat{S}}_{z}^{2}$ can be considered as a sense of fluctuation of magnetic moment, for ${{\rm{\Delta }}}^{2}{\hat{S}}_{z}=\langle {\hat{S}}_{z}^{2}\rangle -{\langle {\hat{S}}_{z}\rangle }^{2}=\langle {\hat{S}}_{z}^{2}\rangle$. It is noted that Δ^2^ *θ* is also equal to the inverse of the classical Fisher information $\mathrm{1/}{\rm{F}}$ through our calculations. Our numerical results of 1/Δ^2^ *θ* are shown in Fig. 4(b), and we can see that 1/Δ^2^ *θ* is robust to different magnetic field intensities, but would be highly reduced when a weak resonant oscillating field is applied. Here it is emphasized that the precision $\mathrm{1/}{{\rm{\Delta }}}^{2}\theta$ for measuring ${\hat{S}}_{z}^{2}$ is one order-of-magnitude larger than that for measuring ${\hat{S}}^{2}$ (i.e., the conventional singlet yield) (see Fig. 4(a) and (b)), and is approximately in the same order as that of QFI (see Figs 1 and 4(b)) especially when $\theta$ is relatively small, which means that the measurement of fluctuation of magnetic moment (i.e., ${\hat{S}}_{z}^{2}$) is close to the optimal one. And the measurement of fluctuation of magnetic moment is also just one of the several potential implementations. In fact, in the spirit of this line, we can judge the feasibility of any possible measurement scheme for avian magnetoreception. For example, the measurement of total magnetic moment of RP is not allowed because its mean value with respect to the RP steady state is equal to 0 for the RP initial state being in the singlet state, such that it can not give any information of the direction of Earth’s magnetic field. However, whether a physically feasible scheme has its biological meaning, just as the singlet yield, needs further behavioral experimental investigations.

### Effect of entanglement and decoherence

Following the present insight that the QFI can well quantify the magnetic sensitivity of RP based compass, it is possible to study the effects of entanglement and different decoherence models on the value of QFI in a unified picture, and we also take the singlet state $|S\rangle$ as the initial state of RP as an example.

**Figure 5:**
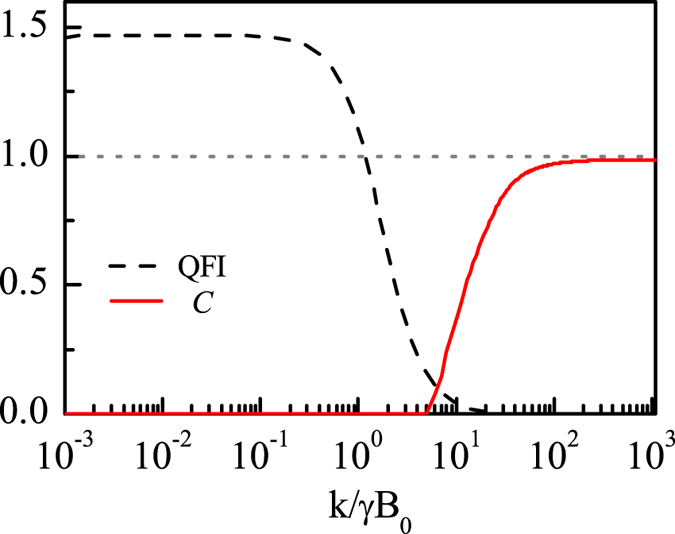
The QFI (black dashed line) and concurrence *C* (red solid line) as a function of the recombination rate *k* for ${{\rm{B}}}_{{\rm{0}}}=46\mu$T and *θ* = *π*/4 with ${A}_{z}=6\gamma \times 46\mu$T, ${A}_{x}={A}_{y}=0$.

Due to the quantum mechanical nature of RP mechanism, the effect of entanglement on the RP based compass has been investigated in terms of singlet yield^13, 14, 16, 19, 20, 30^. Here, we reconsider the effect of entanglement on the RP based compass in terms of QFI, and use concurrence^61^ to quantify entanglement. The concurrence *C* of two qubits is defined as $C({\bar{\rho }}_{s})=\,{\rm{\max }}\{\mathrm{0,\ }{\lambda }_{1}-{\lambda }_{2}-{\lambda }_{3}-{\lambda }_{4}\}$, where ${\lambda }_{i}$ are the square roots of the eigenvalues of the non-Hermitian matrix ${\bar{\rho }}_{s}{\sigma }_{y}\otimes {\sigma }_{y}{\bar{\rho }}_{s}^{\ast }{\sigma }_{y}\otimes {\sigma }_{y}$ arranged in decreasing order, and ${\bar{\rho }}_{s}$ is defined in Eq. (4). As an example, we plot the QFI and $C({\bar{\rho }}_{s})$ as functions of the recombination rate $k$ for ${{\rm{B}}}_{{\rm{0}}}=46\,\mu$T, $\theta =\pi \mathrm{/4}$, ${A}_{z}=6\gamma \times 46\mu$ T, and ${A}_{x}={A}_{y}=0$ with the RP initial state being the singlet state $|S\rangle$ in Fig. 5. And we can see from Fig. 5 that entanglement can not help to promote the performance of RP based compass, which is consistent with the previous work^19^. To be more specific, when *k* is smaller, the QFI is relatively larger which actually corresponds to zero entanglement, and when *k* is larger, the QFI is reduced to zero which corresponds to a relatively larger entanglement instead. It is noted that similar conclusions can be obtained for any other direction angles through our large numerical calculations. In fact, the behavior of entanglement as a function of *k* can be also seen from the expression of ${\bar{\rho }}_{s}$. Specifically, when *k* is small, ${\bar{\rho }}_{s}$ becomes a separable state (see Eq. (S4) of the supplementary materials), which implies that there is no entanglement in ${\bar{\rho }}_{s}$ as shown in Fig. 5. However, when *k* is large, it can be derived from Eq. (S2) and Eq. (S3) of the supplementary materials that ${\bar{\rho }}_{s}$ becomes the singlet state, because in this case the lifetime of RP (~1/*k*) is too short to make a transition between the singlet and triplet states, so the concurrence $C({\bar{\rho }}_{s})$ equals to 1 as shown in Fig. 5.

Besides, in Sec. C of the supplementary materials, we investigate the effects of three typical classes of independent Markovian environmental noise on the value of QFI, namely, the amplitude damping noise, dephasing noise and depolarized noise. By comparing our numerical results with the experimental observations, we find that for the amplitude damping noise and the dephasing noise, the decoherence rate should be smaller than 10 *k*, while for the depolarized noise, the decoherence rate should even be smaller than *k*.

## Discussion

We have investigated the RP based compass from a fully new perspective of QFI. Compared with the conventional approach where the magnetic sensitivity of RP based compass is quantified by the signal contrast ${D}_{s}={{\rm{\Phi }}}_{max}-{{\rm{\Phi }}}_{min}$, our approach that uses the QFI to quantify the magnetic sensitivity proves more accurate and can better reflect the essence of RP based compass. Meanwhile, in this unified approach of QFI, the order of the recombination rate and the effects of entanglement and decoherence on RP based compass can be well understood. Considering that the QFI is only an upper bound of precision for directional detection, it is desirable to seek for a potential measurement scheme to characterize the compass sensitivity. In the spirit of our approach, we have found that the conventional singlet yield is corresponding to the measurement of total angular momentum, which is just one of the several feasible measurement schemes. And the measurement of fluctuation of the total magnetic moment is much more accurate than the singlet yield measurement, and is close to the optimal one. Then an open question naturally arises: among many potential measurement schemes, what is the practical one adopted by birds for orientation? We hope that all these results may help us to understand the mechanism of RP based compass well, which may in turn give us a few clues in the quest to develop quantum technology, and we also expect that the present ideas might be helpful to further apply the concepts and methods developed in quantum information to the field of quantum biology, and gain new insights into the other biological phenomena.

## Methods

Our results are based on the parameter estimation theory. And a standard scenario in quantum parameter estimation can be described as follows: Firstly, a probe system would be prepared in an appropriate initial state $\rho \mathrm{(0)}$, and then it undergoes an evolution which would imprint the parameter information onto the evolved state, say ${\rho }^{x}$, and finally it would subject to a POVM measurement. The overall process is repeated $\nu$ times, and we infer the parameter $x$ from the statistics of the measurement outcomes by choosing an unbiased estimator. The variance of this estimator, i.e., ${{\rm{\Delta }}}^{2}x$, quantifies the error on estimation of *x*, and is lower bounded by:

9 $${{\rm{\Delta }}}^{2}x\ge \frac{1}{\nu {\rm{F}}}\ge \frac{1}{\nu {\rm{QFI}}},$$

where F is the classical Fisher information optimized over all the possible estimators, and QFI is the quantum Fisher information, which is further optimized over all the allowable measurements and is given by ref. 59, 60 and 62

10 $${\rm{QFI}}={\rm{Tr}}[{\rho }^{x}{L}_{{\rho }^{x}}^{2}],$$

where the symmetric logarithmic derivative ${L}_{{\rho }^{x}}$ in the above equation is defined as:

11 $$\frac{d{\rho }^{x}}{dx}\equiv \frac{1}{2}({\rho }^{x}{L}_{{\rho }^{x}}+{L}_{{\rho }^{x}}{\rho }^{x})\mathrm{.}$$

Writing ${\rho }^{x}$ in its spectral decomposition as ${\rho }^{x}={\sum }_{i}{p}_{i}|{\psi }_{i}\rangle \langle {\psi }_{i}|$, one can obtain^62^:

12 $${\rm{QFI}}=2\sum _{{p}_{j}+{p}_{k}\ne 0}\frac{1}{{p}_{j}+{p}_{k}}{|\langle {\psi }_{j}|\frac{d{\rho }^{x}}{dx}|{\psi }_{k}\rangle |}^{2}\mathrm{.}$$

For the RP based compass, the estimated parameter is the geomagnetic field orientation to the basis of HF tensor.

## Electronic supplementary material

Supplementary information
