# Supplementary material for: Quantifying Magnetic Sensitivity of Radical Pair Based Compass by Quantum Fisher Information
Source: Sci Rep. 2017 Jul 19;7:5826. doi: 10.1038/s41598-017-06187-y (PMC5517522; doi:10.1038/s41598-017-06187-y)
Supplement: Supplementary file 1 — Supplementary information [file 41598_2017_6187_MOESM1_ESM.pdf]

Supplementary Materials

Quantifying Magnetic Sensitivity of Radical Pair Based Compass by Quantum Fisher Information

Li-Sha Guo,[1] Bao-Ming Xu,[2] Jian Zou,[1,*] and Bin Shao[1]

[1]School of Physics, Beijing Institute of Technology, Beijing 100081, China

[2]School of Physics, Qufu Normal University, Qufu 273165, China

(Dated: April 26, 2017)

A. Derivation of QFI

In this section, we would derive the approximate expressions of QFI for an arbitrary initial state of RP with and without the oscillating field, i.e., Eq. (6) and Eq. (8) in the main text, respectively. When the horizontal HF coupling $A_x = A_y = 0$, the role of nuclear spin can be considered as applying an effective magnetic field (depending on its state) on the electronic spin. If the nucleus is in the spin up (down) state, the effective magnetic field is $A_z\hat{z}/\gamma(-A_z\hat{z}/\gamma)$, with $\hat{z}$ being the z direction. As a result, the effective Hamiltonian of RP can be written as $H_\pm = \gamma\mathbf{B_0}\cdot(\hat{S}_1+\hat{S}_2) \pm A_z\hat{S}_{2z}$, where $\mathbf{B_0} = \mathrm{B}_0(\sin\theta\cos\phi, \sin\theta\sin\phi, \cos\theta)$ is the geomagnetic field around the RP, with B_0 being the intensity of the geomagnetic field, and θ and ϕ being the orientation of the geomagnetic field to the basis of the HF tensor. The axial symmetry of the HF tensor allows us to set $\phi = 0$ and focus on θ in the range $[0, \pi/2]$ without loss of generality, and θ is the parameter to be estimated for RP based compass. Here we denote the eigenstates of the effective Hamiltonian $H_\pm$ as $|\Psi_\pm^i\rangle \in \{|\phi_1\rangle|\psi_{1\pm}\rangle, |\phi_1\rangle|\psi_{2\pm}\rangle, |\phi_2\rangle|\psi_{1\pm}\rangle, |\phi_2\rangle|\psi_{2\pm}\rangle\}$ and its corresponding eigenvalues as $E_\pm^i$ $(i = 1,2,3,4)$. Specifically, $|\phi\rangle_1 = \cos\frac{\theta}{2}|1\rangle + \sin\frac{\theta}{2}|0\rangle$ and $|\phi\rangle_2 = \sin\frac{\theta}{2}|1\rangle - \cos\frac{\theta}{2}|0\rangle$ are the eigenstates of Hamiltonian of electron 1, i.e., $H_1 = \gamma\mathbf{B_0}\cdot\hat{S}_1$. $|\psi_{1\pm}\rangle = \cos\frac{\theta_\pm}{2}|1\rangle + \sin\frac{\theta_\pm}{2}|0\rangle$ and $|\psi_{2\pm}\rangle = \sin\frac{\theta_\pm}{2}|1\rangle - \cos\frac{\theta_\pm}{2}|0\rangle$ are the eigenstates of Hamiltonian of electron 2, i.e., $H_{2\pm} = \gamma\mathbf{B_0}\cdot\hat{S}_2 \pm A_z\hat{S}_{2z}$, with $\sin\theta_\pm = B_x/B_\pm$, $B_x = \mathrm{B}_0\sin\theta$, $\cos\theta_\pm = (B_z \pm A_z/\gamma)/B_\pm$, $B_z = \mathrm{B}_0\cos\theta$, and $B_\pm = \sqrt{B_x^2 + (B_z \pm A_z/\gamma)^2}$[1,2].

A.1. Derivation of QFI without oscillating field

Given an arbitrary initial state of RP $\rho_s(0)$, we would derive the approximate expression of QFI of the steady state $\bar{\rho}_s$ of RP (see Eq. (4) in the main text) without considering the oscillating field. We can always expand $\rho_s(0)$ in the eigenbasis of the effective Hamiltonian $H_\pm$ as

$$\rho_s(0) = \sum_{i,j=1}^{4} \rho_\pm^{ij}(0)|\Psi_\pm^i\rangle\langle\Psi_\pm^j|, \tag{S1}$$

with $\rho_\pm^{ij}(0) = \langle\Psi_\pm^i|\rho_s(0)|\Psi_\pm^j\rangle$. Generally, the nucleus is initially in a complete mixed state, i.e., $\rho_I(0) = \mathbb{I}/2$. As a result, the state dependent effective magnetic field $A_z\hat{z}/\gamma(-A_z\hat{z}/\gamma)$ induced by the nuclear spin leads to the effective Hamiltonian of RP $H_+(H_-)$ with the same probability 1/2. After some calculations, we can obtain the RP density matrix at time t

analytically:

$$\rho_s(t) = \frac{1}{2}\big(\rho_+(t) + \rho_-(t)\big) \tag{S2}$$

with

$$\rho_\pm(t) = \sum_{i,j=1}^{4} \rho_\pm^{ij}(0) e^{-i(E_\pm^i - E_\pm^j)t} |\Psi_\pm^i\rangle\langle\Psi_\pm^j|. \tag{S3}$$

It has been shown that k should the order of $10^4 s^{-1}$ in different scenarios[2–4], which has also been discussed in the main text (see Fig. 2), so in this paper we let $k = 10^4 s^{-1}$ as well. In this case, $E_\pm^i (10^6 s^{-1} \sim 10^8 s^{-1}) \gg k$, thus the high-frequency oscillating terms of Eq. (S2) have no contribution to the time integral of Eq. (4) in the main text, hence the steady state of RP can be expressed as

$$\bar{\rho}_s \approx \frac{1}{2}\sum_{i=1}^{4} \rho_+^{ii}(0)|\Psi_+^i\rangle\langle\Psi_+^i| + \rho_-^{ii}(0)|\Psi_-^i\rangle\langle\Psi_-^i|. \tag{S4}$$

Now we consider the strong HF coupling approximation, i.e., $A_z \gg \gamma \mathrm{B}_0$, and expand the eigenvectors $|\psi_{1\pm}\rangle$ and $|\psi_{2\pm}\rangle$ in a power series of $\gamma\mathrm{B}_0/A_z$, keeping terms to the first order. Through our calculation, we obtain that $|\psi_{1\pm}\rangle \approx \frac{\gamma \mathrm{B}_0}{2A_z}\sin\theta|1\rangle \mp |0\rangle$ and $|\psi_{2\pm}\rangle \approx |1\rangle \pm \frac{\gamma \mathrm{B}_0}{2A_z}\sin\theta|0\rangle$. Submitting them into Eq. (S4) and keeping terms to the first order of $\gamma\mathrm{B}_0/A_z$, $\bar{\rho}_s$ can be approximately simplified as a diagonal form:

$$\bar{\rho}_s \approx \sum_{i=1}^{2} \rho_1^{ii}|\phi_i\rangle\langle\phi_i| \otimes |1\rangle\langle 1| + \rho_0^{ii}|\phi_i\rangle\langle\phi_i| \otimes |0\rangle\langle 0| \tag{S5}$$

with $\rho_1^{ij} = \langle\phi_i|\langle 1|\rho_s(0)|\phi_j\rangle|1\rangle$, and $\rho_0^{ij} = \langle\phi_i|\langle 0|\rho_s(0)|\phi_j\rangle|0\rangle$. And then according to Eq. (12) in the main text (see Methods), the QFI of $\bar{\rho}_s$ (Eq. (S5)) can be obtained analytically:

$$\mathrm{QFI} \approx \sum_{i=0}^{1} \mathrm{Re}[\rho_i^{12}]^2 \big(\frac{1}{\rho_i^{11}} + \frac{1}{\rho_i^{22}}\big) + \frac{(\rho_i^{11} - \rho_i^{22})^2}{\rho_i^{11} + \rho_i^{22}}, \tag{S6}$$

where $\mathrm{Re}[\rho_i^{12}]$ represents the real part of ρ_i^{12}.

A.2. Derivation of QFI with oscillating field

Now we would derive the approximate expression of QFI of the steady state $\bar{\rho}_s$ (see Eq. (4) in the main text) for an arbitrary initial state of RP with a weak resonant oscillating field $\mathbf{B_{rf}} = \mathrm{B_{rf}}\cos wt(\sin\alpha\cos\beta, \sin\alpha\sin\beta, \cos\alpha)$, where $\mathrm{B_{rf}}$ is the strength of oscillating field with frequency $\omega = 2\gamma\mathrm{B}_0$ being resonant with electron 1. α and β represent the direction of oscillating field with respect to the basis of the HF tensor. Due to the axial symmetry of the

HF tensor we set $\beta = 0$. Here we consider $\alpha = \theta + \pi/2$, namely, the weak oscillating field is perpendicular to Earth's magnetic field. For the convenience of our calculation below, we express an arbitrary initial state of RP $\rho_s(0)$ as

$$\rho_s(0) = \sum_{i,j=1}^{2} \varrho_{\pm}^{ij}(0) \otimes |\psi_{i\pm}\rangle\langle\psi_{j\pm}| \tag{S7}$$

with $\varrho_{\pm}^{ij}(0) = \langle\psi_{i\pm}|\rho_s(0)|\psi_{j\pm}\rangle$ representing the operator of electron 1. Because of the effect of nucleus, the Larmor frequency of electron 2 induced by the effective magnetic field and the geomagnetic field is always not resonant with the frequency of oscillating field, as a consequence, electron 2 can be considered as almost not influenced by the oscillating field[2]. Based on this, the RP density matrix at time t can be obtained as

$$\rho_s(t) = \frac{1}{2}\big(\rho_+(t) + \rho_-(t)\big) \tag{S8}$$

with

$$\rho_{\pm}(t) \approx \sum_{i,j=1}^{2} U(t)\varrho_{\pm}^{ij}(0)U^{\dagger}(t) \otimes e^{-i(\varepsilon_{\pm}^{i} - \varepsilon_{\pm}^{j})t}|\psi_{i\pm}\rangle\langle\psi_{j\pm}|, \tag{S9}$$

where $\varepsilon_{\pm}^{i} = (-1)^{i+1}\gamma B_{\pm}$ $(i = 1, 2)$ are the eigenvalues of $H_{2\pm} = \gamma\mathbf{B_0}\cdot\hat{S}_2 \pm A_z\hat{S}_{2z}$, and $U(t) = \overleftarrow{\mathrm{T}}\exp[-i\int_0^t \mathbb{H}_1(\tau)d\tau]$ is the evolution operator of electron 1 with $\mathbb{H}_1(t) = \gamma(\mathbf{B_0} + \mathbf{B_{rf}})\cdot\hat{S}_1$, and $\overleftarrow{\mathrm{T}}$ denoting the chronological time-ordering operator. After performing the rotating-wave approximation, the evolution operator can be obtained in the eigenbasis $|\phi_i\rangle$ $(i = 1, 2)$ of $H_1 = \gamma\mathbf{B_0}\cdot\hat{S}_1$[5]:

$$U(t) = \begin{pmatrix} \cos\frac{\gamma \mathrm{B_{rf}}t}{2}e^{-i\gamma \mathrm{B_0}t} & i\sin\frac{\gamma \mathrm{B_{rf}}t}{2}e^{-i\gamma \mathrm{B_0}t} \\ \\ i\sin\frac{\gamma \mathrm{B_{rf}}t}{2}e^{i\gamma \mathrm{B_0}t} & \cos\frac{\gamma \mathrm{B_{rf}}t}{2}e^{i\gamma \mathrm{B_0}t} \end{pmatrix}, \tag{S10}$$

When $k = 10^4 s^{-1}$, $\varepsilon_{\pm}^{i} \gg k$, $\gamma\mathrm{B}_0 \gg k$, thus the high-frequency oscillating terms of Eq. (S8) have no contribution to the time integral of Eq. (4) in the main text, hence the steady state of RP under the influence of oscillating field can be expressed as

$$\bar{\rho}_s \approx \frac{1}{2}(\bar{\rho}_+ + \bar{\rho}_-), \tag{S11}$$

with

$$\begin{aligned}\bar{\rho}_{\pm} = & P_{1\pm}|\phi_1\rangle\langle\phi_1| \otimes |\psi_{1\pm}\rangle\langle\psi_{1\pm}| \\ & + P_{2\pm}|\phi_1\rangle\langle\phi_1| \otimes |\psi_{2\pm}\rangle\langle\psi_{2\pm}| \\ & + P_{3\pm}|\phi_2\rangle\langle\phi_2| \otimes |\psi_{1\pm}\rangle\langle\psi_{1\pm}| \\ & + P_{4\pm}|\phi_2\rangle\langle\phi_2| \otimes |\psi_{2\pm}\rangle\langle\psi_{2\pm}|,\end{aligned} \tag{S12}$$

where

$$\begin{aligned}P_{1\pm} = & \varrho^{11}_{\pm}(1,1) + \frac{\gamma \mathrm{B_{rf}} k}{(k^2 + (\gamma \mathrm{B_{rf}})^2)} \mathrm{Im}[\varrho^{11}_{\pm}(1,2)] \\ & - \frac{(\gamma \mathrm{B_{rf}})^2}{2(k^2 + (\gamma \mathrm{B_{rf}})^2)} \big(\varrho^{11}_{\pm}(1,1) - \varrho^{11}_{\pm}(2,2)\big),\end{aligned} \tag{S13}$$

$$\begin{aligned}P_{2\pm} = & \varrho^{22}_{\pm}(1,1) + \frac{\gamma \mathrm{B_{rf}} k}{(k^2 + (\gamma \mathrm{B_{rf}})^2)} \mathrm{Im}[\varrho^{22}_{\pm}(1,2)] \\ & - \frac{(\gamma \mathrm{B_{rf}})^2}{2(k^2 + (\gamma \mathrm{B_{rf}})^2)} \big(\varrho^{22}_{\pm}(1,1) - \varrho^{22}_{\pm}(2,2)\big),\end{aligned} \tag{S14}$$

$$\begin{aligned}P_{3\pm} = & \varrho^{11}_{\pm}(2,2) - \frac{\gamma \mathrm{B_{rf}} k}{(k^2 + (\gamma \mathrm{B_{rf}})^2)} \mathrm{Im}[\varrho^{11}_{\pm}(1,2)] \\ & + \frac{(\gamma \mathrm{B_{rf}})^2}{2(k^2 + (\gamma \mathrm{B_{rf}})^2)} \big(\varrho^{11}_{\pm}(1,1) - \varrho^{11}_{\pm}(2,2)\big),\end{aligned} \tag{S15}$$

$$\begin{aligned}P_{4\pm} = & \varrho^{22}_{\pm}(2,2) - \frac{\gamma \mathrm{B_{rf}} k}{(k^2 + (\gamma \mathrm{B_{rf}})^2)} \mathrm{Im}[\varrho^{22}_{\pm}(1,2)] \\ & + \frac{(\gamma \mathrm{B_{rf}})^2}{2(k^2 + (\gamma \mathrm{B_{rf}})^2)} \big(\varrho^{22}_{\pm}(1,1) - \varrho^{22}_{\pm}(2,2)\big)\end{aligned} \tag{S16}$$

with $\varrho^{ii}_{\pm}(m,n) = \langle\phi_m|\varrho^{ii}_{\pm}(0)|\phi_n\rangle$, $(i,m,n = 1,2)$. Considering the strong HF coupling approximation, i.e., $A_z \gg \gamma \mathrm{B}_0$, Eq. (S11) can be approximately simplified as a diagonal form:

$$\bar{\rho}_s \approx \sum_{i=1}^{2} P^{ii}_1|\phi_i\rangle\langle\phi_i| \otimes |1\rangle\langle 1| + P^{ii}_0|\phi_i\rangle\langle\phi_i| \otimes |0\rangle\langle 0|, \tag{S17}$$

where

$$P^{jj}_i = \rho^{jj}_i + (-1)^j \chi_i \tag{S18}$$

with ρ^{jj}_i having been defined below Eq. (S5), $\chi_i = \frac{(\gamma \mathrm{B_{rf}})^2}{2(k^2+(\gamma \mathrm{B_{rf}})^2)}(\rho^{11}_i - \rho^{22}_i) - \frac{\gamma \mathrm{B_{rf}} k}{(k^2+(\gamma \mathrm{B_{rf}})^2)}\mathrm{Im}[\rho^{12}_i]$ $(i = 0,1,\ j = 1,2)$, and $\mathrm{Im}[\rho^{12}_i]$ represents the imaginary part of ρ^{12}_i. And then according to Eq. (12) in the main text (see Methods), the QFI of $\bar{\rho}_s$ (Eq. (S17)) can be obtained analytically:

$$\mathrm{QFI} \approx \sum_{i=0}^{1} \frac{k^4 \mathrm{Re}[\rho^{12}_i]^2}{(k^2 + (\gamma \mathrm{B_{rf}})^2)^2}\Big(\frac{1}{P^{11}_i} + \frac{1}{P^{22}_i}\Big) + \frac{(P^{11}_i - P^{22}_i)^2}{P^{11}_i + P^{22}_i}. \tag{S19}$$

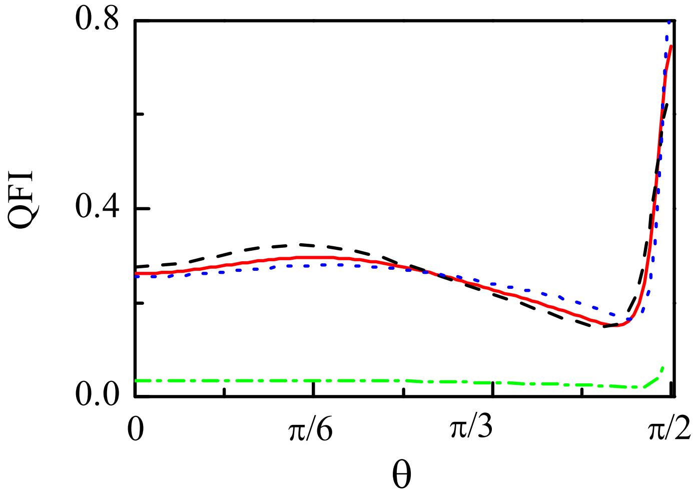

Figure S1. The QFI as a function of the direction angle θ without the oscillating field ($B_0 = 46\mu T$ (red solid line), $B_0 = 59.8\mu T$ (black dashed line), and $B_0 = 32.2\mu T$ (blue dotted line)), and with the oscillating field $B_{rf} = 150nT$ and $B_0 = 46\mu T$ (green dash dotted line). $A_z = 6\gamma \times 46\mu T$, $A_x = A_y = A_z/2$, $k = 10^4 s^{-1}$.

B. QFI with horizontal HF coupling

The effect of external magnetic field and oscillating field on the value of QFI for $A_x = A_y = 0$ has been discussed in the main text. Here we consider the case $A_x = A_y \neq 0$, and calculate the corresponding QFI. In this case, an approximately analytical expression of QFI can not be obtained, thus we calculate the QFI numerically. Through our large numerical calculations, we find that our results are not quite sensitive to what the value of HF coupling is. Here we consider $A_z = 6\gamma \times 46\mu T$, $A_x = A_y = A_z/2$ and the initial state of RP to be the singlet state $|S\rangle$ as an example. The numerical results are shown in Fig. S1, and we can see that the QFI of geomagnetic field ($46\mu T$) is almost not changed when the intensity of magnetic field is decreased ($32.2\mu T$) or increased ($59.8\mu T$) by about 30% of that of geomagnetic field. However, it would be highly reduced when a weak resonant oscillating field perpendicular to Earth's magnetic field is applied. Besides, through our numerical calculations, we find that there is no effect for a weak oscillating field parallel to Earth's magnetic field. These results are similar to that without considering the horizontal HF coupling components in the main text.

C. Effect of decoherence

Decoherence is unavoidable for the RP, and now we reconsider its effect on the RP based compass in terms of QFI. Specifically, we display three typical classes of independent Markovian environmental noises, namely, the amplitude damping noise, dephasing noise and depolarized noise. We describe the environmental noises by the standard Lindblad master equation:

$$\dot{\rho}(t) = -i[H, \rho(t)] + \sum_i \Gamma_i \big(L_i \rho(t) L_i^\dagger - \frac{1}{2}\{L_i^\dagger L_i, \rho(t)\}\big), \tag{S20}$$

where $H = \gamma \mathbf{B} \cdot (\hat{S}_1 + \hat{S}_2) + \hat{I} \cdot \mathbf{A} \cdot \hat{S}_2$ (see Eq. (1) in the main text) denotes the total Hamiltonian for each RP, $\rho(t)$ represents the density matrix of one nucleus and two electrons at time t, Γ_i represents the decoherence rate, $\{\cdot,\cdot\}$ represents the anticommutator, and L_i is the Lindblad operator. For the amplitude damping noise, L_i is only σ_- for each electronic spin individually (i.e., tensored with identity matrices for the nuclear spin and the other electronic spin); for the dephasing noise, L_i is only σ_z for each electronic spin individually; and for the depolarized noise, L_i are σ_x, σ_y, σ_z for each electronic spin individually.

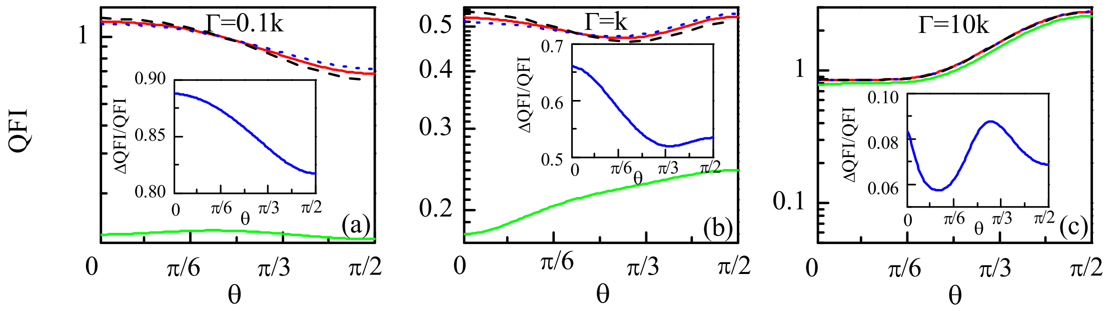

Figure S2. The QFI as a function of the direction angle θ for the amplitude damping noise for (a) $\Gamma = 0.1k$, (b) $\Gamma = k$, and (c) $\Gamma = 10k$ without the oscillating field ($B_0 = 46\mu T$ (red solid line), $B_0 = 59.8\mu T$ (black dashed line), and $B_0 = 32.2\mu T$ (blue dotted line)), and with the oscillating field $B_{rf} = 150nT$ and $B_0 = 46\mu T$ (green dash dotted line). The insets show the corresponding $\Delta QFI/QFI$ with the oscillating field. $A_z = 6\gamma \times 46\mu T$, $A_x = A_y = 0$, $k = 10^4 s^{-1}$.

Firstly, let us examine the effect of uncorrelated amplitude damping noise, with the numerical results shown in Fig. S2. And we can see from Fig. S2(a) that when $\Gamma = 0.1k$, the QFI of 30% weaker and stronger fields are almost not changed compared with that of geomagnetic field. Moreover, a weak resonant oscillating field $B_{rf} = 150nT$, which is

perpendicular to Earth's magnetic field, can highly reduce the QFI with $B_0 = 46\mu T$, and the percent decrease of QFI, i.e., ΔQFI/QFI, can be larger than 80% shown in the inset of Fig. S2(a), which is large enough to imply that a weak oscillating field can completely disrupt the bird. And for $\Gamma = k$ in Fig. S2(b), the 30% stronger and weaker fields still have little influences on the value of QFI. Meanwhile, there still exists an obvious difference in the value of QFI with and without the oscillating field, with ΔQFI/QFI being larger than 50% shown in the inset of Fig. S2(b). However, when $\Gamma = 10k$ in Fig. S2(c), we can see that although the curves of QFI for different magnetic field intensities overlap completely, it would render the bird almost immune to the weak oscillating field, with ΔQFI/QFI being smaller than 10% shown in the inset of Fig. S2(c), which can not account for the fact that a weak oscillating field can completely disrupt the bird. As a conclusion, the decoherence rate should be smaller than $10k$ for this amplitude damping noise.

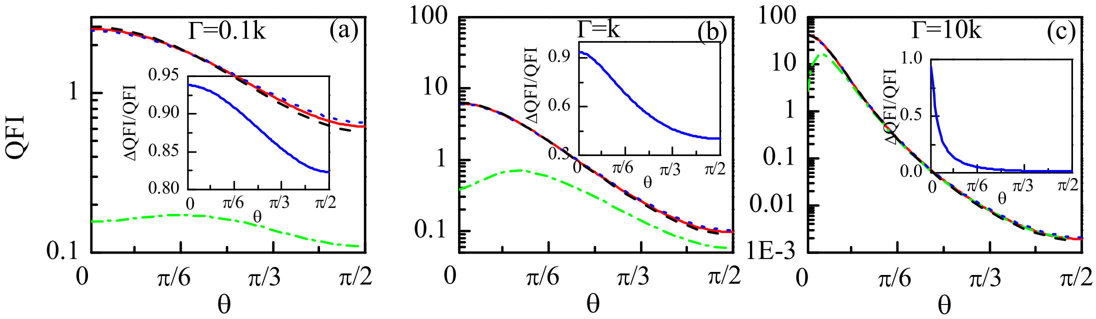

Figure S3. The QFI as a function of the direction angle θ for the dephasing noise for (a) $\Gamma = 0.1k$, (b) $\Gamma = k$, and (c) $\Gamma = 10k$ without the oscillating field ($B_0 = 46\mu T$ (red solid line), $B_0 = 59.8\mu T$ (black dashed line), and $B_0 = 32.2\mu T$ (blue dotted line)), and with the oscillating field $B_{rf} = 150nT$ and $B_0 = 46\mu T$ (green dash dotted line). The insets show the corresponding ΔQFI/QFI with the oscillating field. $A_z = 6\gamma \times 46\mu T$, $A_x = A_y = 0$, $k = 10^4 s^{-1}$.

Next, we consider the effect of uncorrelated dephasing noise, with the numerical results shown in Fig. S3. From Fig. S3 we can see that the 30% stronger and weaker fields than the geomagnetic field have almost no influences on the value of QFI for $\Gamma = 0.1k$, k and $10k$. However, the effects of a resonant oscillating field on the value of QFI are different. Specifically, from Fig. S3(a), we can see that when $\Gamma = 0.1k$, the QFI would be highly reduced when the oscillating field is applied, with ΔQFI/QFI being larger than 80% shown in the inset of Fig. S3(a). And when $\Gamma = k$, the oscillating field is still able to reduce the

value of QFI to some extent, especially when θ is small, ΔQFI/QFI can reach approximately 90% shown in the inset of Fig. S3(b). But when $\Gamma = 10k$, we can see from Fig. S3(c) that the bird becomes quite immune to the weak oscillating field with ΔQFI/QFI being approximately equal to 0 for large θ. Thus for this dephasing noise, the decoherence rate should be smaller than $10k$.

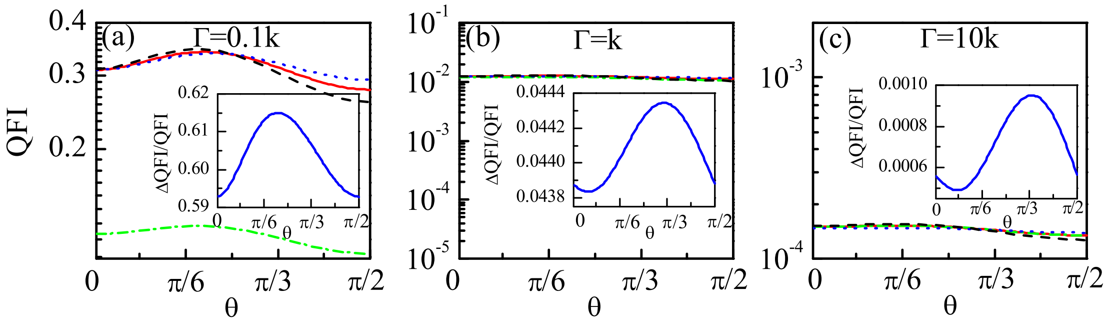

Figure S4. The QFI as a function of the direction angle θ for the depolarized noise for (a) $\Gamma = 0.1k$, (b) $\Gamma = k$, and (c) $\Gamma = 10k$ without the oscillating field ($B_0 = 46\mu$T (red solid line), $B_0 = 59.8\mu$T (black dashed line), and $B_0 = 32.2\mu$T (blue dotted line)), and with the oscillating field $B_{rf} = 150$nT and $B_0 = 46\mu$T (green dash dotted line). The insets show the corresponding ΔQFI/QFI with the oscillating field. $A_z = 6\gamma \times 46\mu$T, $A_x = A_y = 0$, $k = 10^4 s^{-1}$.

Finally, we consider the effect of uncorrelated depolarized noise, with the numerical results shown in Fig. S4. From Fig. S4 we can see that when $\Gamma = 0.1k$, the QFI of 30% weaker and stronger fields are almost unchanged compared with that of geomagnetic field, and the difference in the value of QFI with and without the oscillating field is obvious, with the percent decrease ΔQFI/QFI being larger than 59% shown in the inset of Fig. S4(a). However, when $\Gamma \geq k$, on the one hand, the value of QFI is significantly small despite of its insensitivity to the 30% weaker and stronger fields. On the other hand, the oscillating field has almost no effect on the value of QFI, with ΔQFI/QFI being approximately 4% shown in the inset of Fig. S4(b) or smaller than 0.1% shown in the inset of Fig. S4(c). As a result, for this uncorrelated depolarized noise, the decoherence rate should be smaller than $1k$.

References

---

* Electronic address: zoujian@bit.edu.cn

[1] Cai, J., Caruso, F. & Plenio, M. B. Quantum limits for the magnetic sensitivity of a chemical compass. *Phys. Rev. A* **85**, 040304(R) (2012).

[2] Xu, B. M., Zou, J., Li, H., Li, J. G. & Shao, B. Effect of radio frequency fields on the radical pair magnetoreception model. *Phys. Rev. E* **90**, 042711 (2014).

[3] Gauger, E. M., Rieper, E., Morton, J. J. L., Benjamin, S. C. & Vedral, V. Sustained Quantum Coherence and Entanglement in the Avian Compass. *Phys. Rev. Lett.* **106**, 040503 (2011).

[4] Yang, L. .P, Ai, Q. & Sun, C. P. Generalized Holstein model for spin-dependent electron-transfer reactions. *Phys. Rev. A* **85**, 032707 (2012).

[5] Scully, M. O. & Zubairy, M. S. *Quantum Optics* vol 2 (Cambridge: Cambridge University press) p 151 (1997).
